# Authors’ Response to Peer Reviews of “Human Brucellosis in Iraq: Spatiotemporal Data Analysis From 2007-2018”

**DOI:** 10.2196/60194

**Published:** 2024-07-03

**Authors:** Ali Hazim Mustafa, Hanan Abdulghafoor Khaleel, Faris Lami

**Affiliations:** 1 Department of Inspection, Ministry of Health, Baghdad, Iraq; 2 Surveillance Section, Communicable Diseases Control Center, Public Health Directorate, Ministry of Health, Baghdad, Iraq; 3 College of Medicine, University of Baghdad, Baghdad, Iraq

**Keywords:** human brucellosis, livestock, clustering, spatial, temporal, Iraq

*This is the authors’ response to peer-review reports for “Human Brucellosis in Iraq: Spatiotemporal Data Analysis From 2007-2018.”*

## Round 1 Review

### Anonymous [1]

#### General Comments

*This paper [* *2* ] presents a spatiotemporal distribution analysis of the outbreak of the brucellosis in Iraq from 2007 to 2018, providing explanations for potential underlying causes. The methods employed include descriptive analysis and Getis-Ord G_i_*. The paper exhibits a well-structured format, clear language, rich content, and appropriate methodology.

Response: Thanks for the description.

#### Specific Comments

##### Major Comments

*1. The Abstract and the main text exhibit inconsistency in describing the methods employed. The Results section of the main text only includes the results of the descriptive analysis and Getis-Ord G*_*i*_ *, with no mention of the Moran I method as indicated in the Abstract.

*2. The methods used in the paper should be briefly explained in the Methods section to clarify their principles.*

*3. In the Results section, the authors state that there is an increasing trend in female cases from 2016 onward. This conclusion cannot be drawn; female cases increased from 2016 to 2017 and then decreased by 2018, falling below the 2016 quantity.*

*4. Include spatial distribution maps of the incidence rates for 1-2 years during the study period.*

Response: Maps of 2013 and 2018 were added.

##### Minor Comments

*5. Add numerical labels to the bars in Figure 1 for a more intuitive understanding.*

Response: The numerical labels were added.

*6. Figure 4 lacks coordinate axes, and there is an incomplete gray box on the horizontal axis, affecting aesthetics.*

Response: Figure 4 was revised.

*7. Please provide the formula for calculating the case frequency.*

Response: Thanks for the note. I have added the formula for calculating the incidence per 100,000 as there was no relevant formula for the case frequency—the ones available are math related.

### Anonymous [3]

#### General Comments

*This paper talks about human brucellosis in Iraq and bring an interesting spatiotemporal analysis of the human cases in the country. The paper will contribute to the understanding of human brucellosis in Iraq and can be one more example of the use of spatiotemporal analysis for the control of the disease. However, some changes need to be made to clarify some information in the paper.*

Response: Thanks for the description.

#### Specific Comments

##### Major Comments

###### Introduction

*1. References are missing in the second phrase of the second paragraph.*

Response: Thanks for noticing that. The reference was added.

*2. In the third phrase, “(dogs)” is not necessary.*

Response: Corrected.

*3. The breeding season of sheep and goats is not in spring. So, the phrase “However, it usually coincides with the livestock breeding season, spring” should be changed, as well as “Human exposure to livestock or their contaminated products will occur during spring.” The second part of this phrase is true but not absolute, since animal products can travel to other places; so for clarity, I think this phrase should be reformulated.*

Response: We rewrote the sentence according to the note. However, in Iraq and other parts of Asia, breeding and lambing occur mostly during the spring months (from March to May).

*4. Please, develop this paragraph further: “A study from northern Iraq showed that the prevalence of brucellosis in livestock varied from 1% to 70%, depending on the species and diagnostic methods. 4 Veterinary vaccination program started in 2007. However, its implementation was negatively affected by insecurities in the region.4.” It would be very interesting to know more about the testing and the insecurities of the population regarding vaccination.*

Response: Thanks for the feedback. The prevalence statement was explained further. In addition, the effect of insecurities was also explained.

*5. Please connect more the idea of the first phrase of the fourth paragraph with the following ideas.*

*6. Please define “MOH’s” in the last paragraph.*

Response: Defined.

###### Methods

*7. Please make it clear throughout that the data are about humans and not animals.*

Response: Thanks for the note. We made sure to refer to human brucellosis in all the paragraphs and throughout the manuscript.

*8. In the data description, why does the data come from different sources and with different types of organization and grouping? Can you make it clearer?*

Response: Human brucellosis cases were retrieved from the Surveillance Section at the Ministry of Health. The data were aggregated at the provincial level until 2012. Thereafter, the data became aggregated at the district level.

*9. What is HB? Please define it before using the abbreviation.*

Response: It refers to human brucellosis. We spelled it completely instead of using HB.

*10. What is period one?*

Response: The entire phrase was changed to reflect how data collection at the Surveillance Section changed and how it affected the analysis of the data. The study period from 2007 to 2018 was divided into 2 parts: the first one spanned from 2007 to 2012 when the data were aggregated at the provincial level, and the second part spanned from 2013 to 2018 when the study data were aggregated at the district level.

*11. What does “the low or high attribute zone” mean?*

Response: It means clusters of districts with low or high incidence. The statement was changed to make it clearer.

12. Please explain the P values assumed for the “Getis-Ord Gi* statistic” and what exactly this statistic is identifying.

Response: Thanks for the feedback. A description of the *P* values, its meaning, and interpretation was added to the *Methods*.

*13. In the Abstract, the statistical analysis is explained differently from the Methods. Please make them similar.*

Response: Thanks for the note. The necessary edits were made.

###### Discussion

*14. Have you tested the trend? If so, please clarify the methods and results, but if not, please reformulate the phrase where the word is being used to describe the occurrence of your data.*

Response: Thanks. We reformulated the sentence.

*15. Can you define what were the “ISIL events”?*

Response: It refers to the Islamic State in Iraq and the Levant.

*16. “The number of females has been constantly higher than males.” You are talking about humans, please used woman and man and clarify, in the Methods section, whether this classification was self-made or not.*

Response: Thanks for the constructive feedback. Edits were made in the *Discussion*, *Methods*, and figure.

*17. “housekeeping and farming activities. 5 (11, 12,13).” I did not understand the different configuration of the references in here.*

Response: Corrected.

*18. “However, this age category was very broad and could have been classified into two to three age groups to detect the most commonly affected age group. (14-17).” Why was this change not made? Again, the references are in a strange configuration.*

Response: This change was not made because the data were collected in an aggregated format in these categories and not as a continuous variable that we can regroup.

*19. “The infected animals must be eradicated by slaughtering and burning because there is no curable medical therapy for animal brucellosis.” I did not understand this phrase. Please remove the word eradication from here and explain better why you are saying that the animals should be burned. I do understand that positive animals should be slaughter and their carcasses should be disposed of in the right way, but I have not heard of burning before.*

Response: Thanks for the constructive feedback. Changes were made to reflect the appropriate control methods in animals.

*20. “On the other hand, humans may consume this infected milk unpasteurized, resulting in infection and areas endemic with brucellosis to animals and humans.” Change “to” for “in.”*

Response: Corrected.

*21. There is an “18” in paragraph 5 that could be a reference. I did not understand the phrases that followed the 18.*

Response: We corrected the reference. We changed it to clarify the meaning.

*22. The paragraph “Transboundary transfer of animal brucellosis in the region from the neighboring countries such as Iran, Turkey, and Syria were provoked by war and political instability, lack of immunization and animal quarantine, frequent trading, low awareness and poor knowledge of HB prevention and control, residents with poor sanitary conditions easily exposed to Brucella contaminated food and water sources.” is disconnected from the rest of the text; I did not understand what exactly this is about.*

Response: The entire paragraph was deleted.

*23. The first and second periods of the study are not clear for me.*

Response: This sentence was clarified in the *Methods*.

###### Conclusion

*24. The last part of the conclusion would be better in the Discussion section, such as “Preventive measures such as health education activities should be performed in high-risk areas. Adopting the Quarantine-Slaughter-Immunization strategy and One Health Approach is crucial in controlling the disease. This can be achieved through multisectoral coordination and coordination with neighboring countries in the control programs.”*

Response: Changed.

## Round 2 Review

### Anonymous [1]

#### Specific Comments

##### Major Comments

1. Maybe I did not express it clearly, but for the local Getis-Ord G_i_* method, which is one of the main methods applied in this paper, the authors should give the formula for its calculation and add the source.

Response: Both the formula and the reference were added.

2. This is not a comment that has to be revised. Generally, the significance and spatial location of clusters in the local Getis-Ord G_i_* results are shown on the same map; for example, hot spots with different levels of significance are represented by 3 progressively deeper red colors, and cold spots with different levels of significance are represented by 3 progressively deeper blue colors. Also, Figure 5 contains too many maps, and it is more concise to show the results for 1 year in 1 map.

Response: Thanks for the clarification. We cannot just show the results for 1 map as we are interested in displaying changes over time.

*3. The elements that are really necessary inside a map, including but not limited to a scale, a compass, and preferably the addition of national boundaries, are missing.*

Response: Totally true. However, for the purpose of this study, we are interested in the spatial distribution of human brucellosis and how it changed over time. Adding or removing other complementary features will not affect the results, and adding them may negatively affect the clarity of the map. The borders are, however, displayed.

##### Other Comments

*The authors have finished revising, and I do not have any questions.*

Response: Thanks for your useful feedback.

### Anonymous [3]

#### General Comments

*This paper brings important information and analysis on human brucellosis in Iraq. To improve the paper’s understanding, I suggest an English review of the paper to improve the writing of the paper.*

Response: Thank you for the useful feedback.

#### Specific Comments

##### Major comments

*1. Abstract, section Methods: “The trend of cases by sex and age group were displayed from 2007‐2018 were displayed.” Please delete the last “were displayed.”*

Response: Corrected.

*2. “The seasonal distribution of the cases from 2007 to 2012 was graphed.” Substitute “was” for “were.”*

Response: Corrected.

*3. Introduction: The paragraph on the percentages of occurrence of brucellosis only present the values but does not make a value judgment or interpret what these values mean, why are they important, and so on. Please, reformulate again.*

Response: An explanation was added.

*4. Discussion: Second paragraph: Make it clear that the number of woman you are talking about is the number of woman positive for brucellosis among the analyzed years.*

Response: Corrected.

*5. Conclusion: Substitute HB for human brucellosis.*

Response: Corrected.

## References

[1] Anonymous (2024). Peer review of "Human Brucellosis in Iraq: Spatiotemporal Data Analysis From 2007-2018". JMIRx Med.

[2] Mustafa AH, Khaleel HA, Lami F (2024). Human brucellosis in Iraq: spatiotemporal data analysis from 2007-2018. JMIRx Med.

[3] Anonymous (2024). Peer review of "Human Brucellosis in Iraq: Spatiotemporal Data Analysis From 2007-2018". JMIRx Med.

